# Lrp4/Wise regulates palatal rugae development through Turing-type reaction-diffusion mechanisms

**DOI:** 10.1371/journal.pone.0204126

**Published:** 2018-09-20

**Authors:** Maiko Kawasaki, Katsushige Kawasaki, Fumiya Meguro, Akane Yamada, Ryuichi Ishikawa, Thantrira Porntaveetus, James Blackburn, Yoko Otsuka-Tanaka, Naoaki Saito, Masato S. Ota, Paul T. Sharpe, John A. Kessler, Joachim Herz, Martyn T. Cobourne, Takeyasu Maeda, Atsushi Ohazama

**Affiliations:** 1 Division of Oral Anatomy, Department of Oral Biological Science, Niigata University Graduate School of Medical and Dental Sciences, Niigata, Japan; 2 Centre for Craniofacial Development and Regeneration, Dental Institute, King's College London, Guy's Hospital, London, United Kingdom; 3 Research Center for Advanced Oral Science, Department of Oral Life Science, Niigata University Graduate School of Medical and Dental Sciences, Niigata, Japan; 4 Laboratory of Food Biological Science, Department of Food and Nutrition, Japan Women’s University, Bunkyo, Japan; 5 Department of Neurology, Northwestern University, Feinberg Medical School, Chicago, IL, United States of America; 6 Department of Molecular Genetics, UT Southwestern Medical Center, Dallas, United States of America; Laboratoire de Biologie du Développement de Villefranche-sur-Mer, FRANCE

## Abstract

Periodic patterning of iterative structures is diverse across the animal kingdom. Clarifying the molecular mechanisms involved in the formation of these structure helps to elucidate the process of organogenesis. Turing-type reaction-diffusion mechanisms have been shown to play a critical role in regulating periodic patterning in organogenesis. Palatal rugae are periodically patterned ridges situated on the hard palate of mammals. We have previously shown that the palatal rugae develop by a Turing-type reaction-diffusion mechanism, which is reliant upon Shh (as an inhibitor) and Fgf (as an activator) signaling for appropriate organization of these structures. The disturbance of Shh and Fgf signaling lead to disorganized palatal rugae. However, the mechanism itself is not fully understood. Here we found that *Lrp4* (transmembrane protein) was expressed in a complementary pattern to *Wise* (a secreted BMP antagonist and Wnt modulator) expression in palatal rugae development, representing *Lrp4* expression in developing rugae and *Wise* in the inter-rugal epithelium. Highly disorganized palatal rugae was observed in both *Wise* and *Lrp4* mutant mice, and these mutants also showed the downregulation of Shh signaling, which was accompanied with upregulation of Fgf signaling. *Wise* and *Lrp4* are thus likely to control palatal rugae development by regulating reaction-diffusion mechanisms through Shh and Fgf signaling. We also found that Bmp and Wnt signaling were partially involved in this mechanism.

## Introduction

The genetic commonality of developmental processes is found in many organs. It is believed that they share the same molecular mechanisms and fundamental processes. Periodic patterning is one of the common features observed in many organs, which is believed to develop through general molecular mechanisms. In 1952, Turing proposed a simple model that two morphogens diffusing through a tissue could create self-regulating periodic patterns, the reaction-diffusion model [1]. Simulation of these mechanisms replicates many biological pattern types, such as fish stripes, digits, and feather and hair spacing [2–6]. Turing-type reaction-diffusion mechanisms have thus been shown to play a critical role in regulating periodic patterning in organogenesis [3, 5]. Clarifying the detail of Turing-type reaction-diffusion mechanisms during organogenesis helps to elucidate many other biological processes.

**Fig 1:**
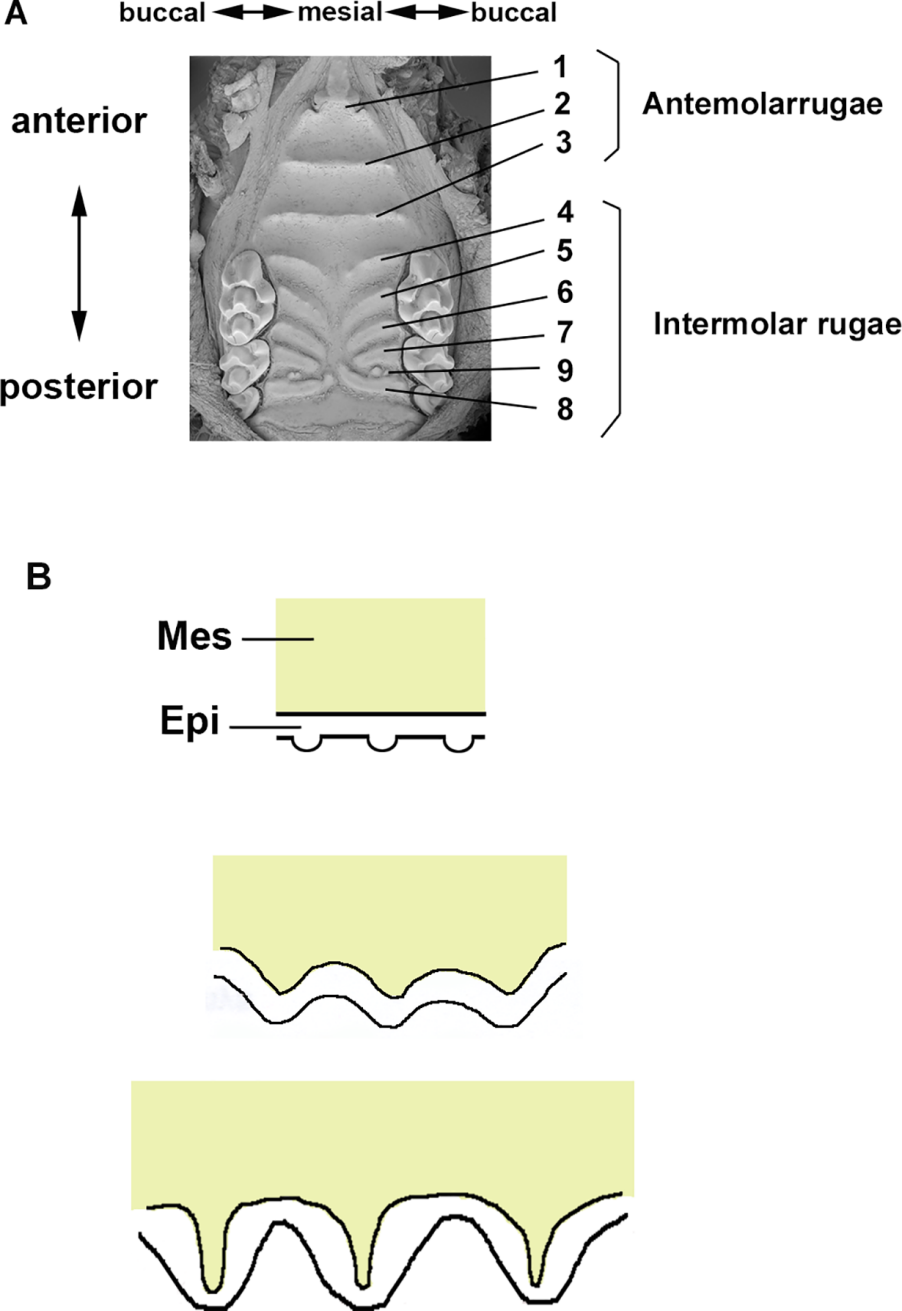
Murine palatal rugae development. (A) SEM image of adult murine palatal rugae. (B) Diagrammatic representation of the developmental stages of palatal rugae formation (sagittal view). Mes; Mesenchyme, Epi; Epithelium.

Palatal rugae are corrugated structures on the hard palate and are conserved in all mammals, including humans, mice and pigs [7–9] They are believed to function in the tactile sensing of objects or food, assisting in holding and crushing food between the tongue and the palate, and aiding in tongue placement during the production of certain speech sounds. There are eight or nine palatal rugae in mice. Three transverse ridges (antemolar rugae) are formed just behind the incisor teeth. The most anterior ruga is fused to the incisive papilla. Five or six rugae (intermolar rugae) are observed between the molar teeth. These rugae are shorter, more oblique and do not cross the midline (Fig 1A). Localized thickening of the palatal epithelium to form placodes is observed as the first morphological sign of rugae development, while the underlying mesenchymal cells condense (Fig 1B) [8]. The outer surface of the epithelium exhibits flat and the thickened epithelium is confined to the inner mesenchymal surface. Then, slight protrusions can be observed on the surface of the developing palatal shelf as the thickened epithelium protrudes over the surface by the flattening of the basement membrane. Subsequently, the placode regions bulge toward the oral cavity to form an overall corrugated appearance [10]. Palatal rugae are sequentially added on the growing palate, their interposition appears to be dependent on activation-inhibition mechanisms, and rugae development has been proposed as a simple tool to study regulation of patterning of serial structures [11]. Although, the number and the patterns of the palatal rugae are species specific, palatal rugae are consistent in mice. Therefore murine palatal rugae development is believed to be under strict genetic control [11–20]. We previously found that Turing-type reaction-diffusion mechanisms are involved in murine palatal rugae development acting through Fgf and Shh signaling [21]; however, the process itself is not fully understood.

The low-density lipoprotein (LDL) receptor family is a large evolutionarily conserved group of transmembrane proteins (for reviews, see [22, 23]). The LDL receptor mainly regulates the concentration of lipoproteins in the extracellular fluids and delivers them to cells (i.e. for the uptake of cholesterol). LDL receptor family members have also been shown to function as direct signal transducers or modulators for a broad range of cellular signaling pathways. Lrp5 and Lrp6 function as co-receptors in the Wnt signaling cascade [24–26]. *Lrp4* (also called *Megf7*) belongs to the LDL receptor family and ENU-induced *Lrp4* null mutants die at birth with defects in the formation of multiple embryonic tissues [27]. We have previously reported that *Lrp4* is involved in regulating tooth development [28, 29]. Wise (also known as USAG-1, Sostdc1 and Ectodin) is a cystine-knot secreted BMP-antagonist, that shows relatively high homology with members of the DAN and CCN peptide families [30]. Wise has also been identified as a context-dependent activator or inhibitor of Wnt signaling by directly binding to Lrp6 [31–33]. Mutation in either of *Wise* or *Lrp4* in mice produces multiple, but identical abnormalities in tooth development linked to alterations in BMP and Wnt signaling [28, 32, 34–40]. We previously reported that physical interaction occurs between Wise and Lrp4 [28].

We report here that *Lrp4* is expressed in a complementary manner to *Wise* expression in murine palatal rugae development, and *Lrp4* and *Wise* mutants display disorganized palatal rugae, which was accompanied by disturbance in Shh and Fgf signaling. *Lrp4*/*Wise* is thus involved in murine rugae development by regulating reaction-diffusion mechanisms.

## Materials and methods

All animal experiments were conducted in compliance with the protocol, which was reviewed by the Institutional Animal Care and Use Committee and approved by the President of Niigata University (Permit Number: #25 Niigata Univ. Res. 255–6).

### Production and analysis of transgenic mice

Production and analysis of transgenic mice *Lrp4* and *Wise* mutant mice were performed as described by Johnson et al. [41] and Kassai et al. [34], respectively. *K14-Bmp4* and *K14-Noggin* mice were produced as described by Guha et al. [42]. *K14-Shh* mice were produced as described by Cobourne et al. [43]. Mouse heads were fixed in 4% paraformaldehyde (PFA), wax embedded and serially sectioned at 7μm. Sections were split over 5–10 slides and prepared for histology or radioactive in situ hybridization. All mice were sacrificed by cervical dislocation.

### *In situ* hybridization

Radioactive *in situ* hybridization with [35S]UTP-labeled riboprobes and whole-mount *in situ* hybridization with DIG-labeled riboprobes was carried out as described previously by Ohazama et al. [28, 44]. *In situ* hybridization was performed in triplicate.

### Immunohistochemistry analysis

After deparaffinization of sections, tissues were treated with proteinase K and then incubated with an antibody to Phosphorylated-Smad 1/5/9 (Cell signaling Technology). As a negative control, normal rabbit serum or normal goat serum were used instead of primary antibody. Tyramide signal amplification system was performed (Parkin Elmer Life Science) for detecting phosphorylated-Smad 1/5/9. Slides were mounted with Aquamount. Pictures were taken with the same exposure between control, wild-type, Wise and Lrp4 mutant mice. Immunohistochemistry analysis was performed in triplicate.

### Whole-mount nuclear fluorescent imaging

Detailed morphology of palatal rugae was analyzed by nuclear fluorescent imaging technique, called “Pseudo SEM” as previously described [45]).

## Results

### *Lrp4* and *Wise* expression in palatal rugae development

**Fig 2:**
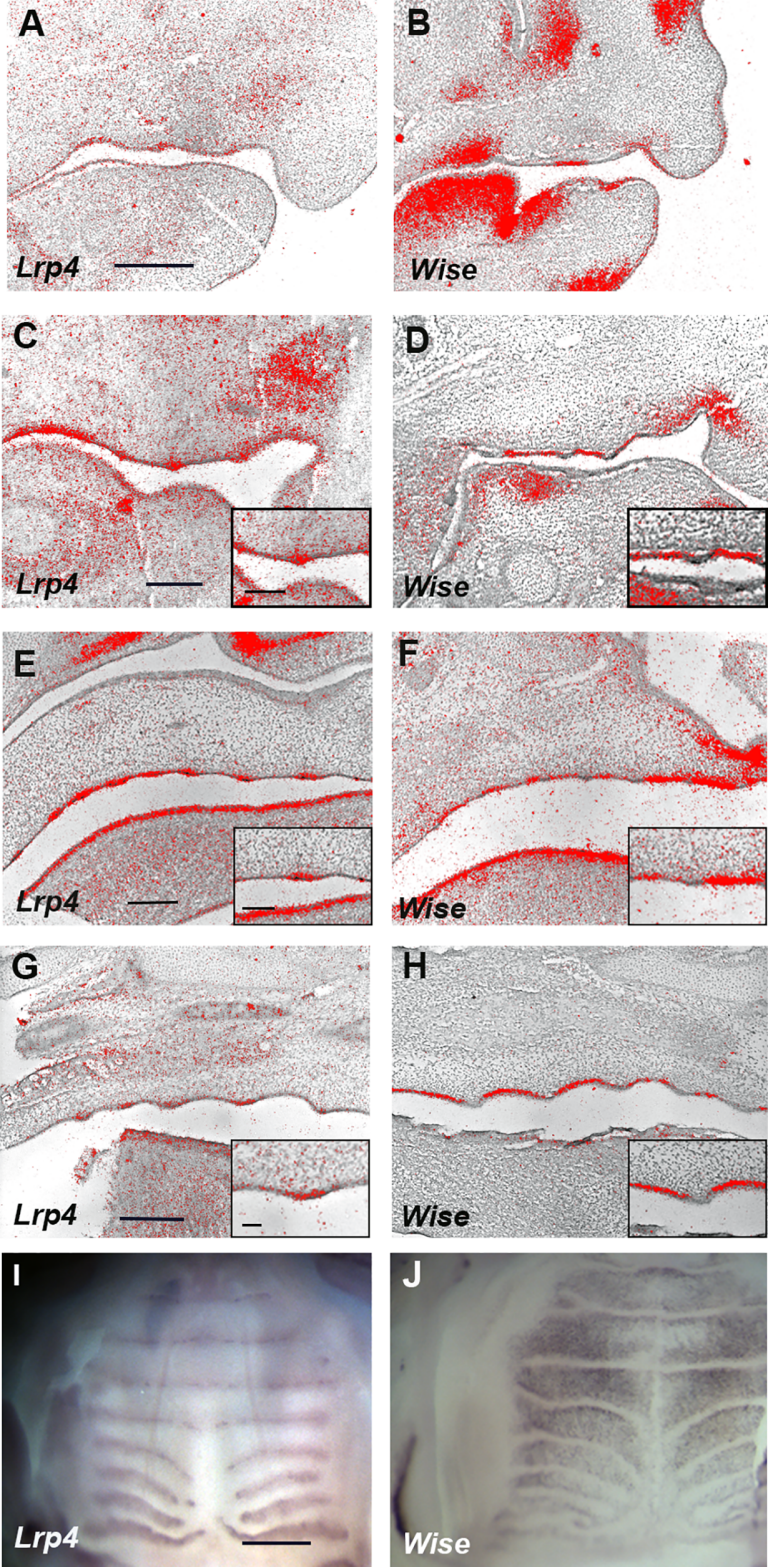
Expression of *Lrp4* and *Wise* during palatal rugae development. (A-H) Sagittal sections of developing palate between midline and molar region showing *in situ* hybridization of *Lrp4* (A, C, E, G) and *Wise* (B, D, F, H) in wild-type at E12.5 (A, B), E13.5 (C, D), E14.5 (E, F) and E16.5 (G, H). (C-H) Box showing large magnification images of developing rugae region. (I, J) Whole mount in situ hybridization of *Lrp4* (I) and *Wise* (J) in wild-type palatal rugae at E14.5. Scale bar; 100μm (box in C-H), 200μm (A-D), 300μm (E, F), 400μm (G-J).

**Fig 3:**
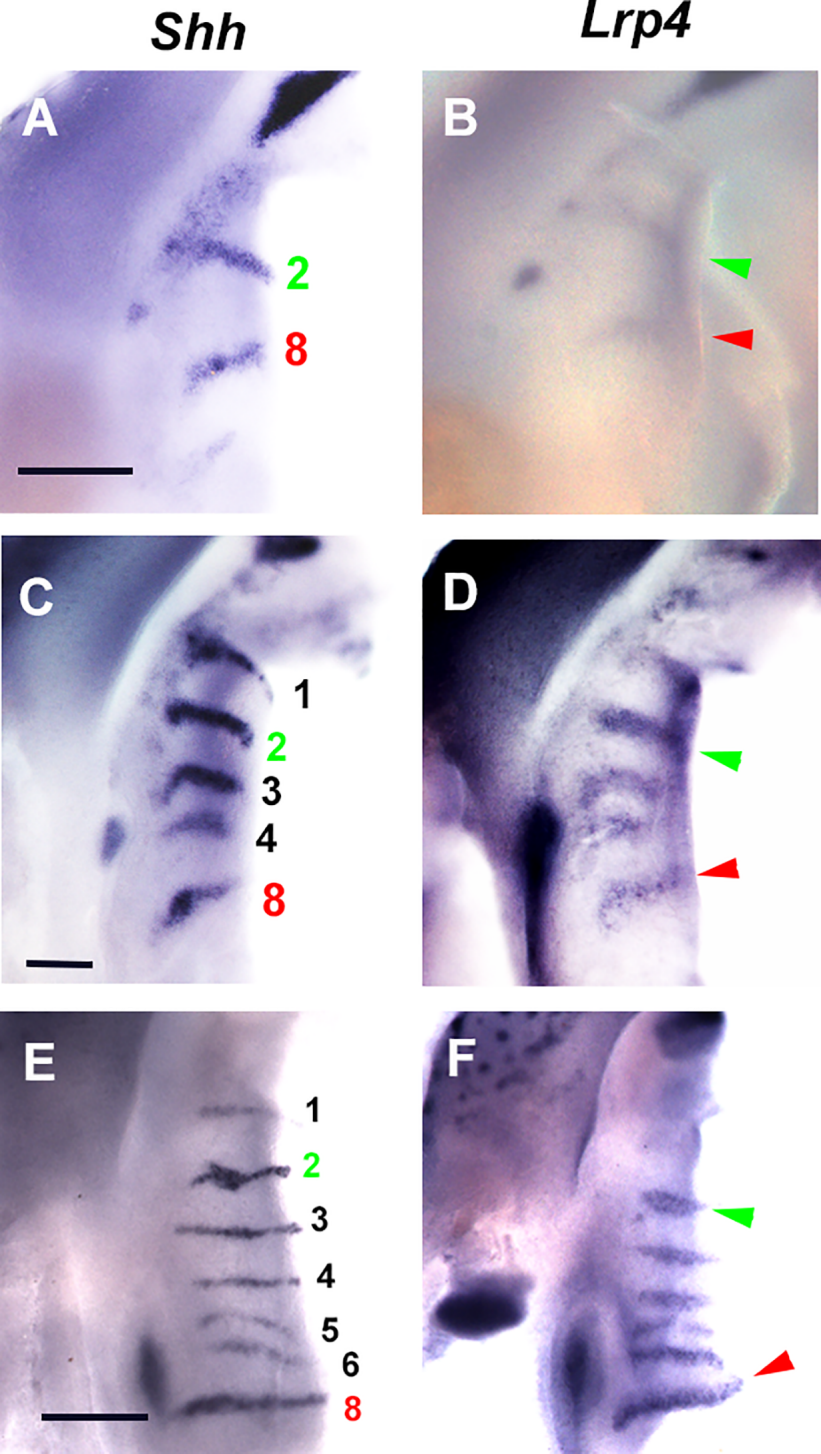
*Lrp4* expression during sequence of rugae appearance. Oral views of palatal rugae showing expression of *Shh* (left palatal shelf) and *Lrp4* (right palatal shelf) in wild-type. Left and right panel showing developing palatal shelf obtained from same embryo. Green and red arrowheads indicating region corresponding ruga 2 and 8, respectively. Scale bar; 200μm (A-D), 400μm (E, F). A, B; E11.5-E12.0, C, D; E12.5-E13.0, E, F; E13.5-E14.0. Images of *Lrp4* expression were horizontally flipped.

**Fig 4:**
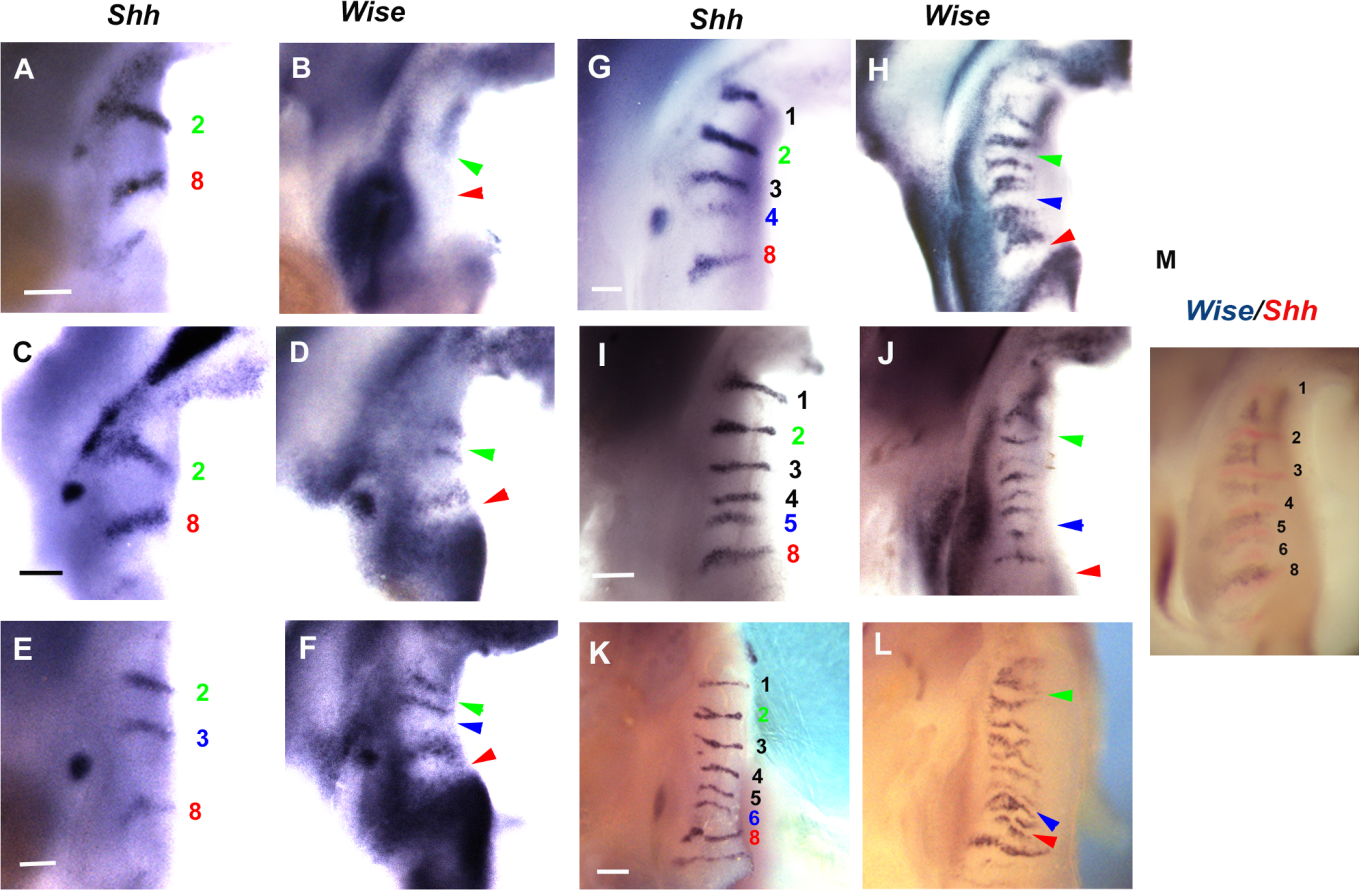
*Wise* expression during sequence of rugae appearance. (A-L) Oral views of palatal rugae showing expression of *Shh* (left palatal shelf) and *Wise* (right palatal shelf) in wild-type. Green and red arrowheads indicating region corresponding ruga 2 and 8, respectively. Left and right panel showing developing palatal shelf obtained from same embryo. Blue arrowheads indicating region corresponding newly formed ruga. Scale bar; 200μm (A-H), 400μm (I-L). (M) Oral views of palatal rugae showing expression of *Shh* (red) and *Wise* (blue) in wild-type. A-D; E11.5-E12.0, E, F; E12.0-E12.5, G, H; E12.5-E13.0, I, J; 13.0-E13.5, K, L, M; E13.5-E14.0. Images of *Wise* expression were horizontally flipped.

**Fig 5:**
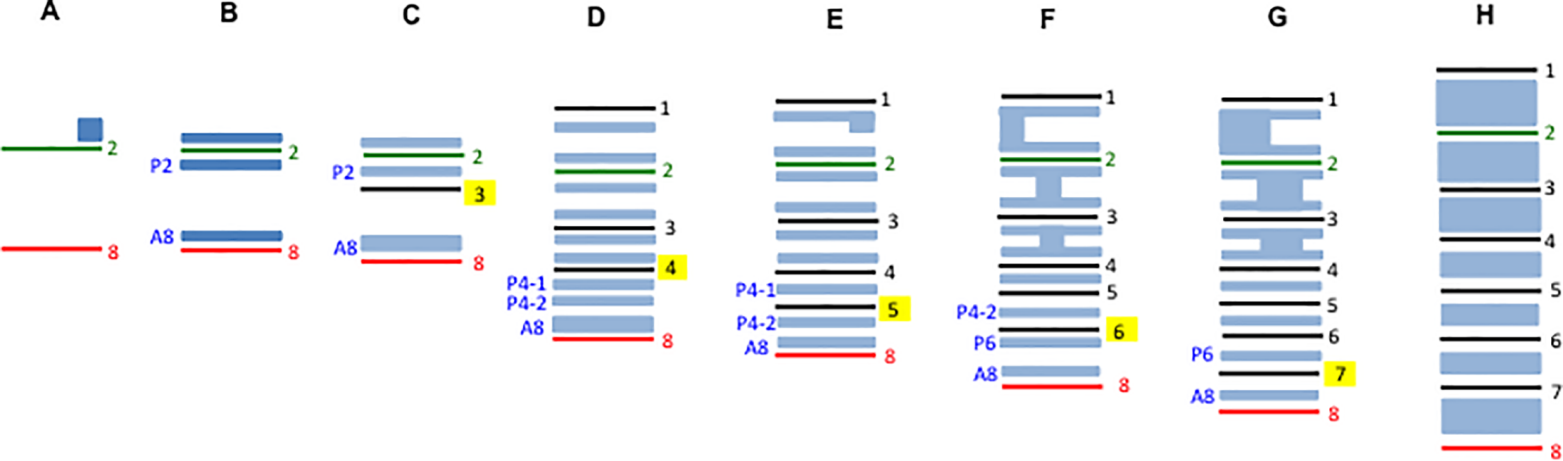
*Wise* expression during palatal rugae development. Diagrammatic representation of *Wise* expression during the developmen of palatal rugae. Light blue representing *Wise* expression. A, B; E11.5-E12.0, C; E12.0-E12.5, D; E12.5-E13.0, E; 13.0-E13.5, F; E13.5-E14.0, G; E14.5–15.0, H;16.0-E16.5. P2; *Wise* expression domain posterior to ruga 2, A8; *Wise* expression domain anterior to ruga 8, P4-1; *Wise* expression domain posterior to ruga 4, P4-2; *Wise* expression domain between P4-1 and A8 *Wise* expression domain, P6; *Wise* expression domain posterior to ruga 6.

In order to clarify the detail of Turing-type reaction-diffusion mechanisms during palatal rugae development, we were interested in *Lrp4* and *Wise*. It is known that both molecules interacted each other in organogenesis such as tooth and mammary development [28, 29, 40]. Disorganized patterns of palatal rugae have previously reported in *Wise* mutant mice [15]. We found that *Lrp4* was expressed in a complementary manner to *Wise* expression in palatal rugae development from days of gestation (E) 12.5 to E16.5 (Fig 2). Radioactive *in situ* hybridization analysis exhibited that *Wise* and *Lrp4* were expressed in the epithelium during all the examined developmental stages. *Lrp4* expression was observed in the developing rugae, whereas *Wise* was expressed in the inter-rugal epithelium during palatal rugae development. It is known that palatal rugae are sequentially added to the developing palate [11, 14]. To understand how *Lrp4* and *Wise* were expressed in the sequential addition of rugae in murine rugae development, we performed the whole mount *in situ* hybridization of *Lrp4* or *Wise* and *Shh* (as the marker of palatal rugae) on the left and right palatal shelves from same embryo, respectively. While *Shh* expression were observed in the regions corresponding to ruga 2 and 8 (E11.5-E12.0), *Lrp4* was only weakly expressed in the same region (Fig 3A and 3B). Timing and localization of *Lrp4* expression were similar to those of *Shh* during the sequential appearance of rugae (Fig 3C–3F). While *Shh* expression was observed in the regions corresponding to ruga 2 and 8 (E11.5-E12.0), *Wise* was expressed in the region anterior to ruga 2 alone (Figs 4A, 4B and 5A). Soon after, *Wise* was expressed in the regions anterior and posterior (*Wise* expression domain posterior to ruga 2; P2) to ruga 2, and in the region anterior to rugae 8 (*Wise* expression domain anterior to ruga 8; A8), while *Shh* was expressed in regions corresponding ruga 2 and 8 only (Figs 4C, 4D and 5B). Subsequently (E12.0-E12.5), *Shh* was expressed in the region posterior to P2 as ruga 3 (Figs 4E, 4F and 5C). While *Shh* was expressed as ruga 4 (E12.5-E13.0), *Wise* was expressed in the regions anterior and posterior to ruga 3 (Figs 4G, 4H and 5D). *Wise* was also expressed as two domains in the region posterior to developing ruga 4 (*Wise* expression domain posterior to ruga 4; P4-1, *Wise* expression domain between P4-1 and A8 domain; P4-2; Figs 4H and 5D). *Wise* expression was also observed in the region anterior to developing ruga 4 (Figs 4H and 5D). *Shh* expression as ruga 5 was observed between P4-1 and P4-2 *Wise* expression domains (Figs 4I, 4J and 5E; E13.0-E13.5). While *Shh* expression (ruga 6) was observed in region posterior to P4-2 *Wise* expression domain (E13.5-E14.0), *Wise* was also expressed at the region posterior to forming ruga 6 (*Wise* expression domain posterior to ruga 6; P6; Figs 4K, 4L and 5F). Meanwhile, *Wise* expression between ruga 1 and 2, ruga 2 and 3, and ruga 3 and 4 began to fuse (Figs 4K, 4L and 5F). The expressions of *Shh* and *Wise* were also confirmed by double whole mount *in situ* hybridization (Fig 4M). *Shh* (ruga 7) was then expressed in the region between the P6 and A8 *Wise* expression domain (Fig 5G, E14.5-E15.0). *Wise* expression between rugae then expanded. *Wise* was, therefore, expressed through two mechanisms: expansion from a single-expression domain and fusion of two expression domains. Interestingly, the former was observed in the intermolar rugae region and the latter in the antemolar rugae.

### Palatal rugae phenotypes in *Lrp*4 and *Wise* mutant mice

**Fig 6:**
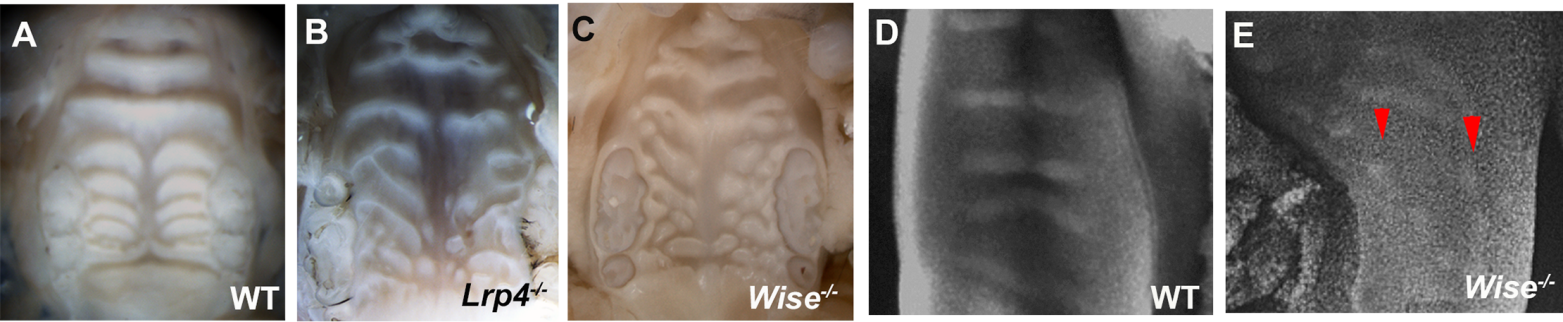
Palatal rugae phenotypes in *Lrp4* and *Wise* mutant mice. Oral views of palatal rugae in adult wild-type (A), *Lrp4* (B) and *Wise* mutants (C). (D, E) Pseudo SEM image of palatal rugae of wild-type (D) and *Wise* mutant (E) mice at E13.5-E14.0. Arrowheads indicating rugae.

**Fig 7:**
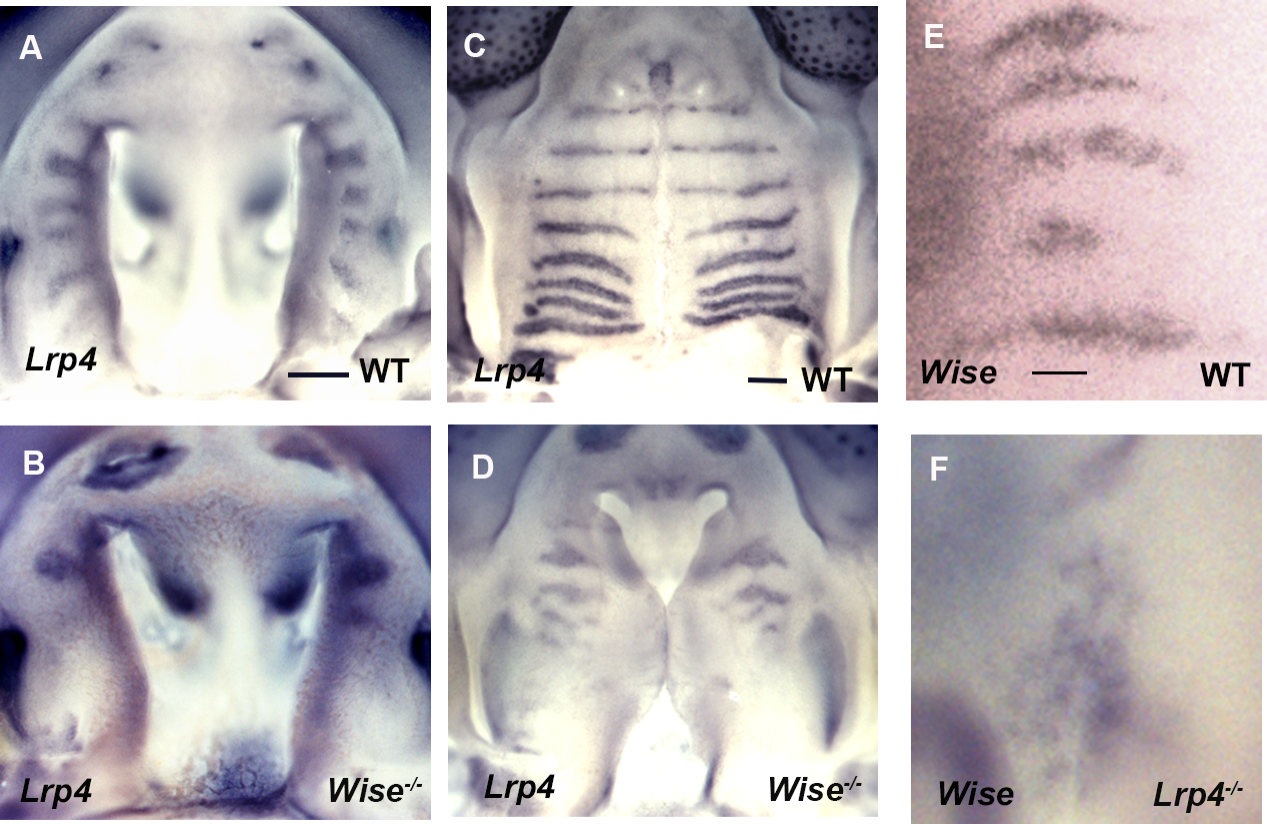
*Wise* and *Lrp4* expression in *Lrp4* and *Wise* mutant mice. Whole mount explants showing *Lrp4* (A-D) and *Wise* (E, F) transcription in palatal rugae of wild-type (A, C, E), *Wise* (B, D) and *Lrp4* (F) mutant mice at E13.5-E14.0 (A, B, E, F) and E14.0-E14.5 (C, D). Scale bar; 200μm (A-D), 50μm (E, F).

In order to investigate the role of *Lrp4* and *Wise* in palatal rugae development, palatal rugae were examined in *Lrp4* and *Wise* mutant mice. Both mutants showed disorganized pattern of palatal rugae as full penetrance, which differed in each mutant mouse (Fig 6B and 6C, *Lrp*4 mutant mice: N = 20/20, *Wise* mutant mice: N = 35/35). In both mutants, antemolar rugae exhibited minor disorganized pattern, whereas intermolar rugae was highly disorganized. These suggested that *Lrp4* and *Wise* were essential molecules for palatal rugae development, especially for intemolar rugae. After the initiation of rugae, ruga growth extended toward the midline in wild-type mice (Fig 6D) [8]. The arrest of rugae extension, ectopic initiation of rugae and the changing of the direction of ruga growth were observed during mutant rugae development (Fig 6E, data not shown). To determine whether there is any interaction between *Lrp4* and *Wise* during the palatal rugae development, we examined *Lrp4* and *Wise* expression in *Wise* and *Lrp4* mutant mice, respectively. *Lrp4* expression was significantly reduced in *Wise* mutant mice, particularly in the intermolar region, while *Wise* expression was disorganized in *Lrp4* mutants (Fig 7A–7F). In addition to other ectoderm-derived organs including mammary gland and tooth [28, 29], *Lrp4* and *Wise* were thus likely to be interact each other to regulate palatal ruage development.

### Shh and Fgf signaling in palatal rugae of *Lrp4* and *Wise* mutant mice

**Fig 8:**
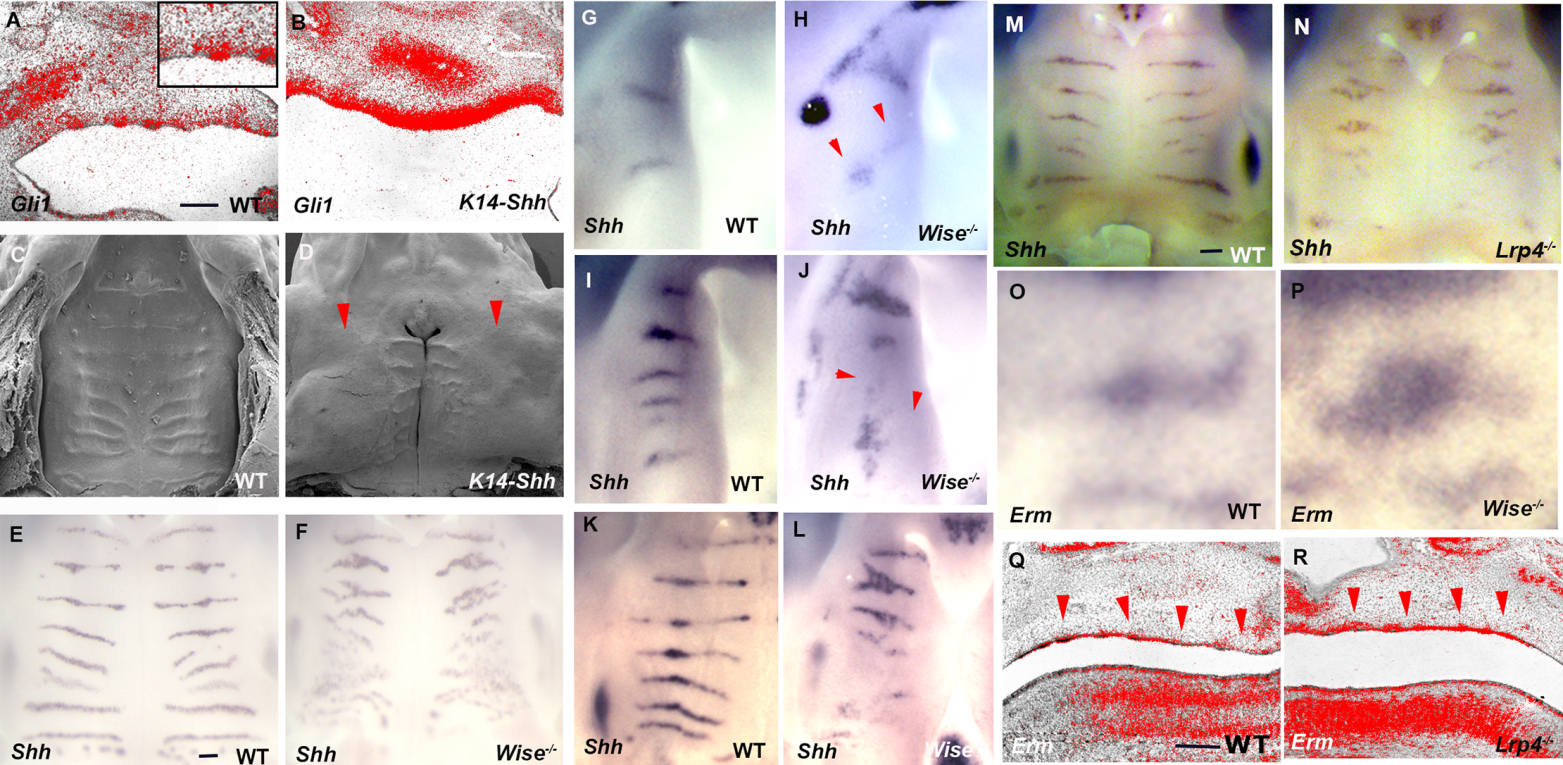
Shh and Fgf signaling in *Lrp4* and *Wise* mutants during palatal rugae development. (A, B, Q, R) Sagittal sections of developing palate between midline and molar region showing *in situ* hybridization of *Gli1* (A, B) and *Erm* (Q, R) in wild-type (A, Q), *Shh-K14* (B) mice and *Lrp4* mutant mice (R) at E13.5. Arrowheads indicating *Erm* expression in developing palatal rugae. (A) Box showing large magnification images of developing rugae region. (C, D) SEM image of palatal rugae of wild-type (C) and *Shh-K14* (D) mice at birth. Arrowheads indicating palatal rugae presumptive region. (E-P) Whole mount explants showing *Shh* (E-N) and *Erm* (O, P) transcription in palatal rugae of wild-type (E, G, I, K, M, O), *Lrp4* (N) and *Wise* (F, H, J, L, P) mutant mice at E12.5 (G, H), 13.5 (I, J, O, P), E14.0 (K, L) and E14.5 (E, F, M, N). Scale bar; 200μm.

Shh has been shown to act as an inhibitor in Turing-type reaction-diffusion mechanisms during palatal rugae development [21]. In fact, overexpression of *Shh* in palatal epithelium resulted in reduced palatal rugae (Fig 8B and 8D). *Shh* expression was found to be significantly downregulated in the intermolar rugae in *Wise* mutant mice at E14.5 (Fig 8F). A slight alteration in *Shh* expression was observed in *Wise* mutant mice at E12.5, when ruga2 and 8 were formed (Fig 8H). Subsequently, the downergulation of *Shh* expression was evident in mutant palates (Fig 8J and 8L). *Shh* expression was also downregulated in *Lrp4* mutants (Fig 8N). In Turing-type reaction-diffusion mechanisms during palatal rugae development, Fgf signaling was previously identified as an activator [21]. Conversely to Shh signaling, expression of *Erm*, a marker of Fgf signaling, was expanded in developing palatal rugae of both *Wise* and *Lrp4* mutants at E13.5 (Fig 8P and 8R). The abnormal palatal rugae found in *Wise* and *Lrp4* mutants was thus likely to be involved in the disturbance of Turing-type reaction-diffusion mechanisms. These also suggested that *Wise* and *Lrp4* mutants were excellent experimental models for investigating the detail of Turing-type reaction-diffusion mechanisms during palatal rugae development.

### Bmp signaling in palatal rugae development

**Fig 9:**
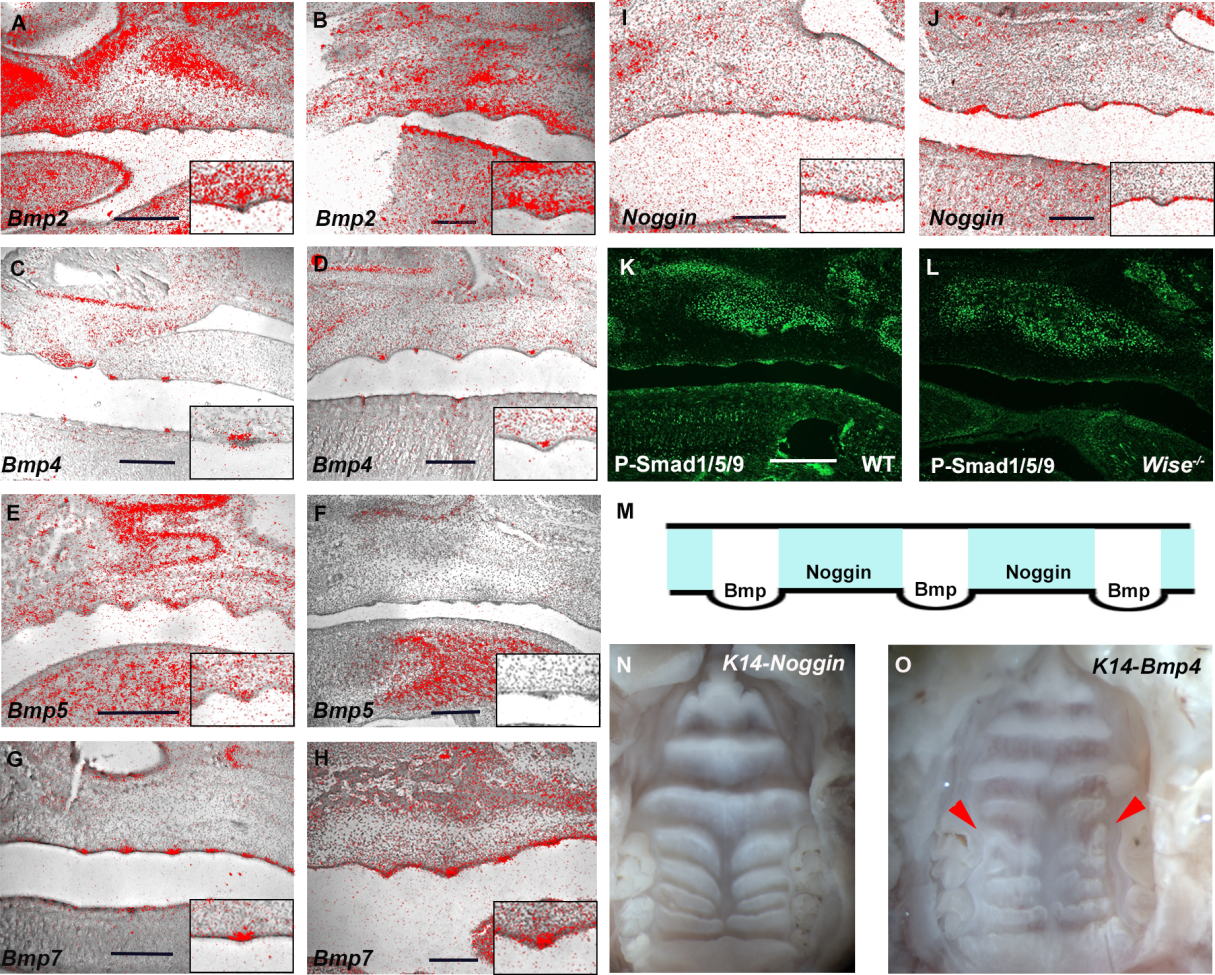
Bmp signaling in palatal rugae development. (A-L) Sagittal sections of developing palate between midline and molar region showing *in situ* hybridization of *Bmp2* (A, B), *Bmp4* (C, D), *Bmp5* (E, F), *Bmp7* (G, H) and *Noggin* (I, J) at E14.5 (A, C, E, G, I) and E16.5 (B, D, F, H, J) and immunohistochemistry of p-Smad1/5/9 (K, L) in wild-type (A-K) and *Wise* mutants (L) at E13.5. Box showing large magnification images of developing rugae area. (M) Schematic of Bmp signaling activity and *Noggin* expression in palatal rugae in sagittal view. Oral views of palatal rugae in adult *Noggin-K14* (N) and *Bmp4-K14* mice (O). Red arrowheads indicate disorganized rugae. Scale bar; 200μm.

*Lrp4* and *Wise* are involved in Bmp signaling in tooth development [28, 34, 36–40]. In order to investigate whether Bmp signaling is involved in palatal rugae development, we firstly examined the expression of major Bmp ligands in the rugae development. *Bmp2*, *Bmp4* and *Bmp5* were expressed in mesenchyme underneath the developing rugae, whilst *Bmp7* expression was found in the developing rugae at E14.5 and E16.5 (Fig 9A–9H). In addition to ligands, expression of the Bmp inhibitor *Noggin*, was found in the inter-rugal epithelium (Fig 9I and 9J). To identify where Bmp signaling is activated in rugae development, immunohistochemistry for phosphorylated Smad1/5/9 (p-Smad1/5/9) was performed in the developing palatal rugae. P-Smad1/5/9-positive cells were observed in developing rugae, suggesting that Bmp signaling is activated in developing rugae, but not in the inter-rugal region where *Noggin* was expressed (Fig 9K). Bmp signaling is thus likely to be controlled by the balance between ligands and inhibitors (Fig 9M). P-Smad1/5/9 immunolocalization showed downregulation in *Wise* and *Lrp4* mutants (Fig 9L, data not shown). In order to investigate the role of Bmp signaling in palatal rugae development, we also examined the palatal rugae of mice overexpressing *Bmp4* (*K14-Bmp4*) or *Noggin* (*K14-Noggin*) under K14 promoter. Both *K14-Bmp4* and *K14-Noggin* mice showed only minor anomalies in the palatal rugae. *K14-Noggin* mice showed only five intermolar rugae, while six are usually observed in wild-type at this stage (Figs 6A and 9N). Only slightly disorganized palatal rugae were observed in *K14-Bmp4* mice (Fig 9O, *K14-Bmp4* mice:N = 7/7, *K14-Noggin* mice: N = 9/9).

### Wnt signaling in palatal rugae development of *Lrp4* and *Wise* mutant mice

**Fig 10:**
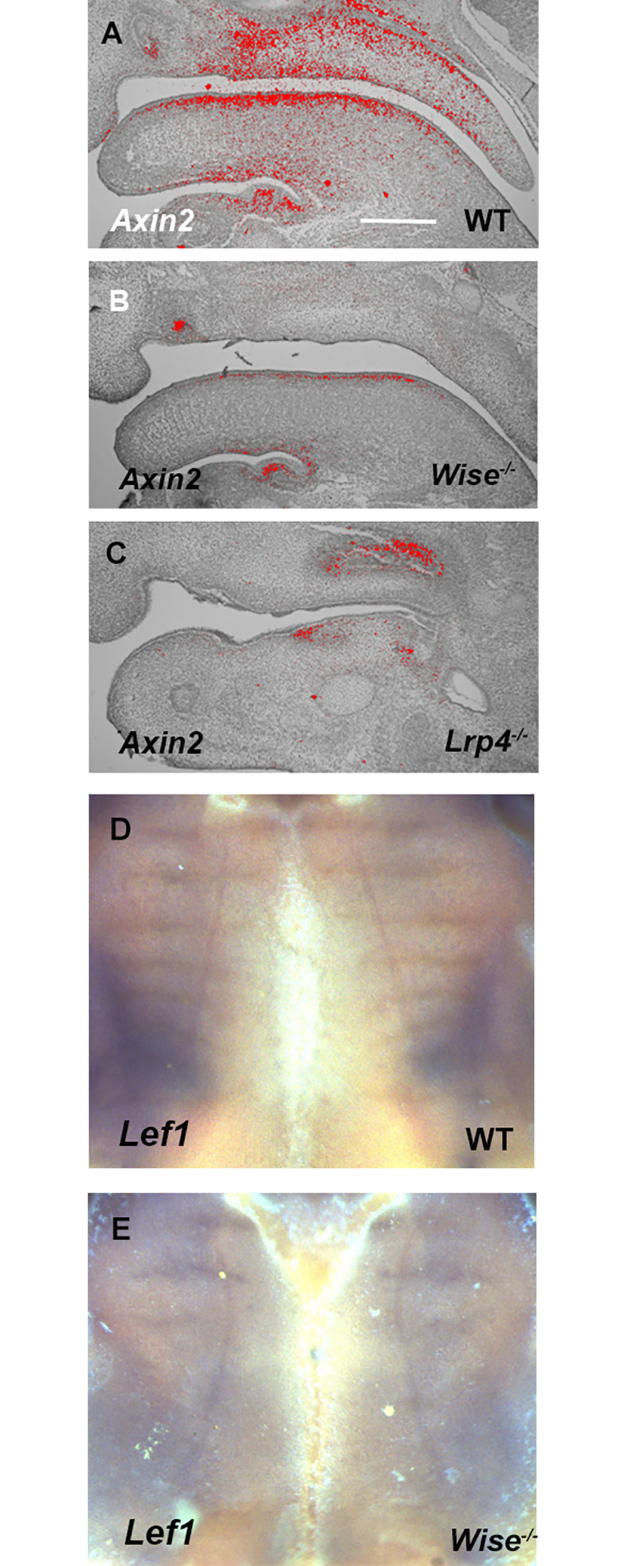
Wnt signaling in *Lrp4* and *Wise* mutant mice. (A-C) Sagittal sections of developing palate at region between midline and molar showing *in situ* hybridization of *Axin2* in wild-type (A), *Wise* (B) and *Lrp4* (C) mutants at E14.5. Scale bar; 200μm. (D, E) Whole mount explants showing *Lef1*transcription in palatal rugae of wild-type (D) and *Wise* (F) mutant mice.

*Lrp4* and *Wise* are also known to be involved in Wnt signaling in tooth development [28, 34, 36, 37, 39]. *Lef1* and *Axin2* have been shown to be expressed in palatal rugae development [18]. In order to examine the changes of canonical Wnt signaling in *Lrp4* and *Wise* mutant embryos, we examined the expression of *Lef1* and *Axin2* (a marker of canonical Wnt signalling). Both mutants showed downregulation of *Axin2* expression at E14.5 (Fig 10A–10C). *Lef1* expression was also reduced in mutants, particularly in the intermolar rugae (Fig 10E).

## Discussion

Wise can physically bind to Lrp4, and *Lrp4* is expressed in a complementary pattern to *Wise* expression in tooth development. Furthermore, tooth phenotypes are identical between *Lrp4* and *Wise* mutants, suggesting that *Lrp4* interacts with *Wise* during tooth development [28, 29, 34, 36, 37, 39]. Similarly, *Lrp4* and *Wise* expression was found to be complementary during palatal rugae development (Figs 2–5), and the phenotypes of palatal rugae were comparable between *Lrp4* and *Wise* mutants (Fig 6). *Lrp4* is thus highly likely to interact with *Wise* during palatal rugae development.

We found that palatal rugae development is controlled by a Turing-type reaction-diffusion mechanism through Fgf and Shh signaling morphogens. Disturbance of Shh and Fgf signaling has been shown to lead to disorganized palatal rugae, which were identical to those of mice with altered Shh and Fgf signaling [21]. We have previously shown that the disturbance of the reaction-diffusion mechanism by applying inhibitor of Shh signaling led to the expansion of Fgf response makers in palatal rugae development [21]. In *Wis*e and *Lrp4* mutants, the downregulation of Shh signaling was accompanied by the expansion of Fgf signaling (Fig 8). This suggested that disorganized palatal rugae in the *Wise* and *Lrp4* mutants is likely to be caused by the disturbance of Turing-type reaction-diffusion mechanisms through Fgf and Shh signaling. *Wise* and *Lrp4* mutant mice are thus excellent experimental model for investigating the detail of Turing-type reaction-diffusion mechanisms during palatal rugae development.

*Lrp4* and *Wise* expression are found in a complementary pattern in both tooth and rugae development. *Wise* expression is observed in tooth mesenchyme, while *Lrp4* is expressed in tooth epithelium, suggesting that correlation between *Lrp4* and *Wise* is through epithelial-mesenchymal interaction. However, unlike their expression in tooth development, both *Wise* and *Lrp4* were expressed in palatal epithelium, with *Lrp4* in the developing rugae and *Wise* in the inter-rugal epithelium. Our results also showed that Bmp signaling activity and Bmp inhibitor expression are found in a complementary pattern in palatal rugae development, with activity in the epithelial placode, and inhibition in the inter-placode epithelium. We found that palatal rugae development is under the control of Turing-type reaction-diffusion mechanisms through Fgf and Shh signaling morphogens [21]. *Shh* and *Erm* were also expressed in palatal rugae epithelium [15, 16]. Additionally, hair follicle spacing has been shown to be determined by reaction-diffusion mechanisms using morphogens expressed in the epithelium [5]. Interaction between developing rugae and inter-rugal epithelium thus plays a critical role in palatal rugae development. No significant changes in *K14-Bmp4* and *K14-Noggin* mice, suggesting that *Bmp* and *Noggin* might be able to be compensated by other molecules during palatal rugae development. It is possible that there is an interaction between *Wise* and *Noggin* in the inter-rugal epithelium, and between *Lrp4*, *Shh* and *Bmp* in developing rugae.

Our work, in addition to observations by other groups, indicates that *Lrp4* and *Wise* are strongly linked to Bmp signaling in tooth development [28, 34, 36, 37, 39]. We found that Bmp signaling was downregulated in developing palatal rugae of both mutants (Fig 9L). However, the disturbance of Bmp signaling resulted in only minor anomalies during palatal rugae development. The alteration of Bmp signaling due to *Lrp4*/*Wise* mutation thus weakly influenced palatal rugae development, suggesting that the down-regulation of Bmp signaling is not a major cause of abnormal palatal rugae formation in *Wise* and *Lrp4* mutants. We could not exclude the possibility that the minor rugae phenotypes observed in *K14-Bmp* and *K14-Noggin* mice are related to inter-strain variability. In addition to Bmp signaling, *Lrp4* and *Wise* are strongly related to Wnt signaling in tooth development [28, 34, 36, 37, 39]. Downregulation of Wnt signaling was observed in the palatal rugae of both *Lrp4* and *Wise* mice (Fig 10). It has been shown that Fgf and Shh pathways are major downstream targets of Wise-regulated Wnt signaling in tooth development [38]. In addition, Shh signaling has been shown to be downstream of Wnt signaling in palatal rugae development [18]. It is conceivable that there is feedback loop between Shh and Wnt signaling in palatal rugae (i.e. the lack of Shh lead to the downregulation of Wnt signaling). It is also possibile that Wise/Lrp4 is directly interacted with Shh and Fgf signaling and the alteration of Bmp and Wnt signaling was consequence of the changes of Shh and Fgf signaling. On the other hand, it has been shown that none of Wnt signaling results in no palatal rugae formation, indicating that Wnt signaling determines the initiation of palatal rugae which contain regulating palatal rugae patterning [18]. Therefore, we could not exclude the possibility that reduced Wnt signaling allow rugae to form, but affect patterning of rugae in *Wise* and *Lrp4* mutants.

Rugae disorganization was more severe in the intermolar rugae subset, compared to those of the antemolar rugae in *Lrp4* and *Wise* mutants (Fig 6B and 6C). Changes of Shh signaling in mutants were also found to be different between antemolar and intermolar rugae (Fig 8). *K14-Bmp4* and *K14-Noggin* also showed different phenotypas of palatal rugae between antemolar and intermolar rugae (Fig 9N and 9O). We also found that the processes of establishment in *Wise* expression also differed between antemolar and intermolar rugae (Figs 4 and 5). The palatal rugae show periodic patterning, but the pattern is slightly different between the anterior and posterior region; the antemolar rugae are transverse ridges, and the intermolar rugae are shorter, more oblique and do not cross the midline. It is likely that molecular mechanisms in rugae development is slightly difference between regions.

Periodic patterning of iterative structures, the palatal rugae, develops by Turing-type reaction-diffusion mechanisms. Our results also indicate that the intricate molecular network is involved in the palatal rugae development, although two morphogens diffusing through a tissue finally create self-regulating periodic patterns.
